# Single-step plasma-induced hierarchical structures for tunable water adhesion

**DOI:** 10.1038/s41598-019-56787-z

**Published:** 2020-01-21

**Authors:** Tae-Jun Ko, Sang Jin Park, Min-Sung Kim, Sun Mi Yoon, Seong Jin Kim, Kyu Hwan Oh, Sahn Nahm, Myoung-Woon Moon

**Affiliations:** 1 Life and Materials Science Research Division, Korea Institute of Science and Technology, Seoul, 02792 Republic of Korea; 2 Department of Materials Science and Engineering, Seoul National University, Seoul, 08826 Republic of Korea; 3 Department of Materials Science and Engineering, Korea University, Seoul, 02841 Republic of Korea

**Keywords:** Wetting, Polymers

## Abstract

Smart surfaces in nature have been extensively studied to identify their hierarchical structures in micro-/nanoscale to elucidate their superhydrophobicity with varying water adhesion. However, mimicking hybrid features in multiscale requires complex, multi-step processes. Here, we proposed a one-step process for the fabrication of hierarchical structures composed in micro-/nanoscales for superhydrophobic surfaces with tunable water adhesion. Hierarchical patterns were fabricated using a plasma-based selective etching process assisted by a dual scale etching mask. As the metallic mesh is placed above the substrate, it serves the role of dual scale etching masks on the substrate: microscale masks to form the micro-wall network and nanoscale masks to form high-aspect-ratio nanostructures. The micro-walls and nanostructures can be selectively hybridized by adjusting the gap distance between the mesh and the target surface: single nanostructures on a large area for a larger gap distance and hybrid/hierarchical structures with nanostructures nested on micro-walls for a shorter gap distance. The hierarchically nanostructured surface shows superhydrophobicity with low water adhesion, while the hybrid structured surface becomes become superhydrophobic with high adhesion. These water adhesion tunable surfaces were explored for water transport and evaporation. Additionally, we demonstrated a robust superhydrophobic surface with anti-reflectance over a large area.

## Introduction

Hierarchical structures appearing in nature with various special functions have recently been investigated, such as lotus leaf’s superhydrophobicity^1–3^, Fish scale’s underwater superoleophobicity^4,5^, moth eye’s anti-reflectivity^1,6,7^, gecko toes’ dry adhesion, and butterfly wing’s photonic crystals^8^. These functionalities are expressed by the constituents of the chemical substance as well as the multiscale structures from the molecular level to the macro-structure on the surface harnessed for their living environment^9–11^. In particular, the superhydrophobic surfaces with water contact angle (CA) exceeding 150° are present in nature, such as lotus leaf, rose petal, and peanut leaf^11–16^. They have hierarchical or hybrid structures in micro-/nanoscale to enhance the high static water CA, while the surface adhesions to water are diverse to allow for it to adjust to its surrounding environment; lotus leaf has low water adhesion and high mobility of water droplet, thus leading to a self-cleaning property, while peanut leaf and rose petal have a high adhesion and low mobility to water, which are beneficial for water harvesting or evaporation control^17,18^.

The water adhesion of these superhydrophobic surfaces has been reported to depend on the shape and scale of the three-phase contact line (TCL), which is composed of water/solid/air. Research has revealed that, as the TCL becomes discontinuous by lowering the contact area due to micro or nanoscale features and the scale of individual TCL becomes smaller, the air-pocket between the structures becomes stable, decreasing the water adhesion^19,20^. Conversely, certain surfaces form the high water adhesion, even at high CAs, with water penetrating through the surface cavities to increase the contact area, ultimately resulting in the formation of the continuous TCL^18,21–23^. These findings underscore the importance of appropriately selecting the required structure and its smart combination, such as the hierarchical or hybridized structures, to control the adhesion of the superhydrophobic surfaces.

To date, various methods such as spray coating^24^, femtosecond laser^25^, electrospinning^26^, and electrodeposition^27^ have been used to fabricate well-defined micro/nanostructures on the desired surfaces with tunable adhesion. However, most of these methods require multiple-step procedures to mimic nature’s smart architecture in multiscale from nano to macro or its hybridization. Also, previous approaches have a limitation on realizing large-area surface patterning with high uniformity. Recently, a plasma-based selective etching method has been used to fabricate superhydrophobic surfaces with high-aspect-ratio nanostructures^28–30^. The nanostructures could be fabricated on carbon- or silicon-based materials *via* selective plasma etching with the metal co-deposition, and the morphology of the nanostructure could be tuned through plasma parameters such as the gas mixing ratio, chamber pressure, treatment duration, or co-deposited metal species^31^. In the primary mechanism of nanofabrication through selective ion etching, metal atoms sputtered from the cathode plate are co-deposited on the target substrate. These metal atoms can aggregate and form clusters on the substrate, forming a region with high etch resistance and low reactivity to the plasma. However, other regions exposed to plasma can be etched at a high etch rate. This anisotropic or preferential etching leads to the formation of nanostructure with high-aspect-ratio on the substrate during the continuous plasma etch with reactive gases such as O_2_, CF_4_, and SF_6_^29^. This template-less method is a time-saving and eco-friendly process for the large-scale fabrication of nanostructures with tunable structural shapes such as dot, pillar, or hairy^32,33^. However, there are some limits to applying this method in large-scale fabrication, as the formation of the etching mask from the supply of the co-deposited metal atoms may not be uniform over a large surface area. In addition to that, as the etch mask size governed by the co-deposited metal atoms or its clusters is within a few tens of nanometers, the etched features are only formed in nanoscale, so the fabrication of patterns in micro/macroscale or hybridized micro/nano-scale is necessary for the additional complex processes such as wet etching or soft lithography^34^.

**Figure 1:**
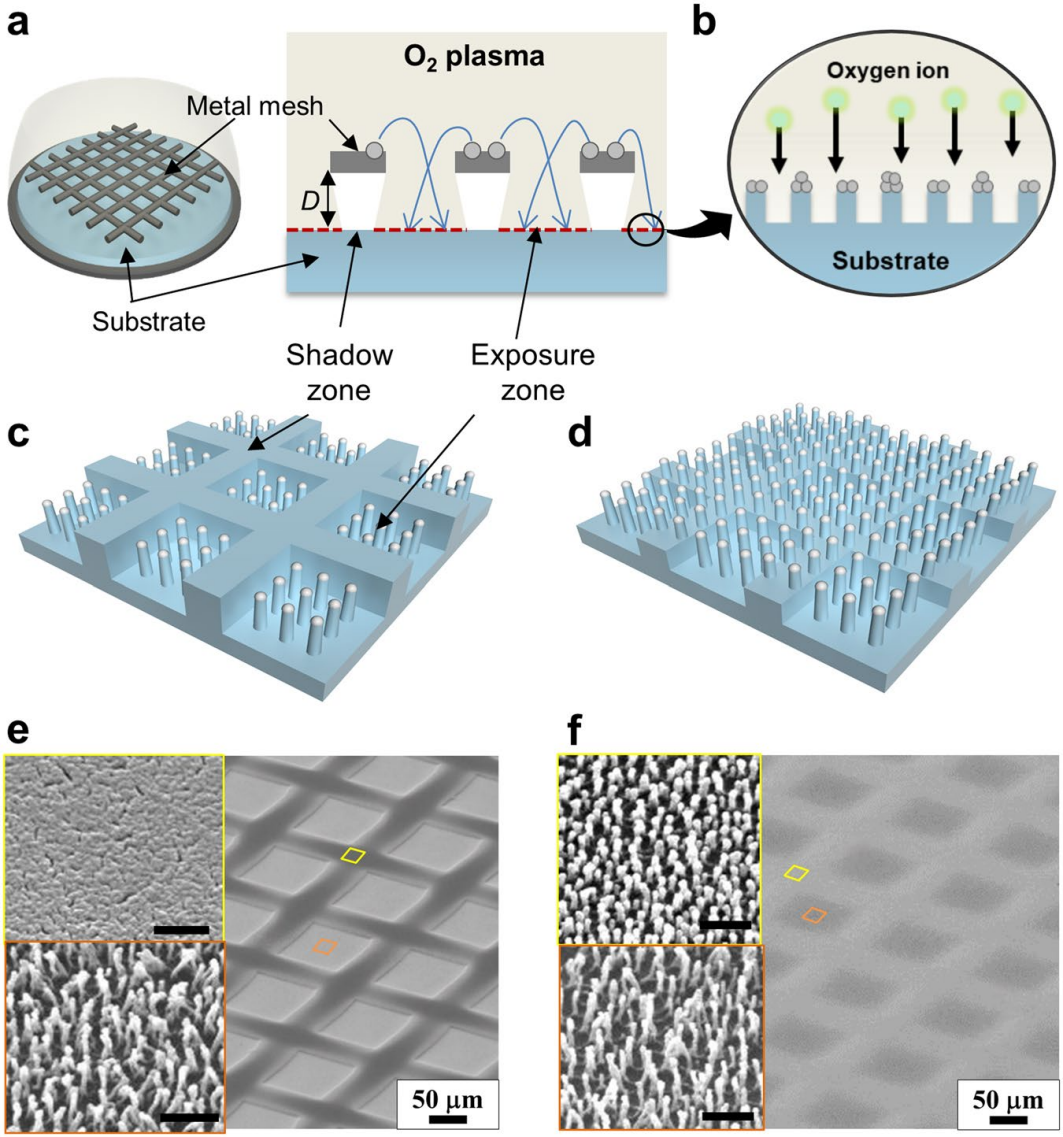
Illustrations of hybrid patterning process assisted with metal-mesh overhung showing two characteristic etching masks; (**a**) microscale shadow zone formation, and (**b**) nanoscale metal etch masks formation. (**c**–**f**) Schematics and corresponding SEM images showing (**c**,**e**) hybrid patterned surface consisting of the flat micro-wall and high-aspect-ratio nanostructures, and (**d**,**f**) hierarchical patterned surface with low micro-wall and nanostructures. Scale bars in insets represent 500 nm.

Here, we proposed a one-step process for the fabrication of hierarchical structured superhydrophobic surfaces with tunable water adhesion through the plasma-based selective ion etching assisted by the dual-scale etching mask. By placing the metallic mesh with wire diameters and spaces in a few hundreds of micrometers directly above the polymeric substrate, the dual-scale etching masking effect was realized by controlling the gap distance between the substrate and metallic mesh. In the single-step process, under plasma exposure in a glow discharge of oxygen gas, the metallic mesh serves as two characteristic etching masks on the substrate: a microscale etching mask to form a micro-wall network by creating a plasma shadow zone on the substrate (Fig. 1a) and a nanoscale etching mask to form nanostructures with a high aspect ratio through the selective ion etching mechanism (Fig. 1b). By tuning the gap distance (*D*), the nanostructures and micro-walls can be easily hierarchized, as the plasma ion can reach under the shadow zone with respect to *D*. For the small *D*, simple hybrid structures were formed with the nanostructures surrounded by the micro-wall network (Fig. 1c,e), while well-defined hierarchical structures were formed with the nanostructures nested on the entire surfaces of the micro-wall pattern for the larger gap distance (Fig. 1d,f).

In this paper, the fabrication process of hierarchical structures was first discussed with varying gap distances. Then, the suggested patterning with the dual-scale etching mask effect was compared with the conventional plasma-based selective ion etching. The results showed that after the hydrophobic coating, the hybrid structured surface showed superhydrophobicity with high water adhesion, while the hierarchically structured surfaces superhydrophobicity with low adhesion. Two contrasting conditions of water adhesion were applied for water transport and evaporation. Further, it was demonstrated that optical reflectance could be reduced through the formation of well-distributed nanostructures on the large area substrate.

## Results

### Microscale etching mask for hierarchical structuring

**Figure 2:**
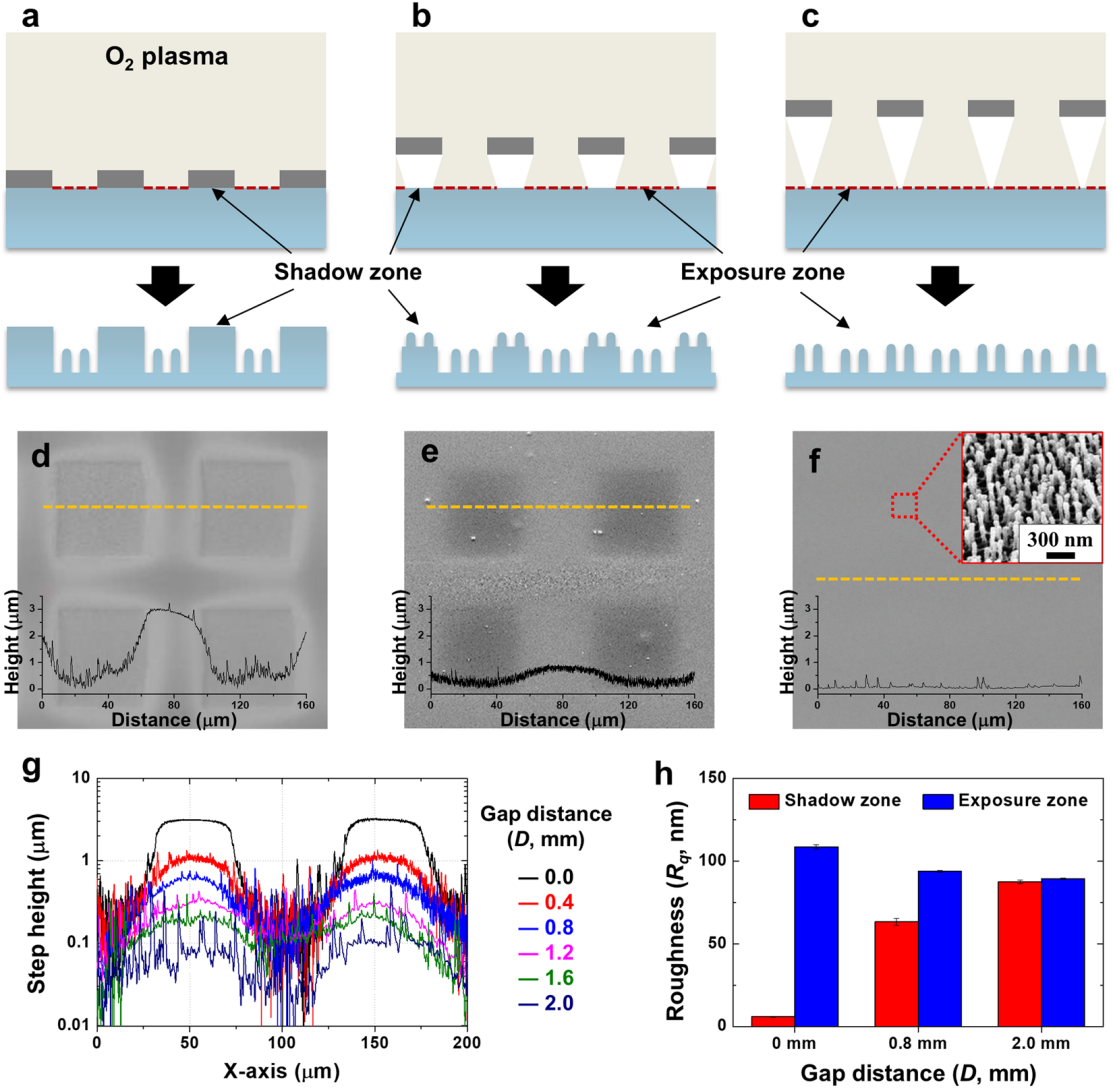
(**a**–**c**) Illustrations of hybrid patterning process assisted by metal-mesh with varying gap distances (*D*) between the mesh and the substrate; (**a**) *D* = 0.0 mm, (**b**) 0.8 mm, and (**c**) 2.0 mm. (d-f) SEM images and AFM line profiles of O_2_ plasma-treated PET substrates with various *D*; (**d**) *D* = 0.0 mm, (**e**) 0.8 mm, and (**f**) 2.0 mm, respectively. (**g**) The AFM line profile with respect to various gap distances. (**h**) Comparison of roughness between the region under mesh (shadow zone) and the region under mesh hole (exposure zone) with respect to various *D*.

Figure 2a–c illustrate the flow of reactive oxygen ions when the metal mesh is placed on the polyethylene terephthalate (PET) substrate with varied gap distance in the plasma system. According to the principle of the plasma etcher system, the reactive ions that are biased by electrode would accelerate toward the substrate placed on the cathode plate^29^. In our system, the metal mesh placed on the substrate acted as a mask to block the oxygen ions to reach the sample surface underneath the metal mesh fiber. Therefore, the area of the substrate exposed by the hole of the mesh was exposed to oxygen ions which caused reactive ion etching. Whereas the area of the substrate under the metal fiber of the mesh could not be etched because oxygen ions were blocked. Thus, the masked area by metal fiber, which was not etched out, formed the microscale wall. Depending on the gap distance, areas of exposure zone and shadow zone were varied as shown in Fig. 2a–c.

When the metal mesh is in direct contact with the substrate (*D* = 0.0 mm), as shown in Fig. 2a, the oxygen ions only reached the exposure zone, while the shadow zone directly under the metal wire was blocked from plasma exposure. Thus, the micro-wall patterns were formed with the well-defined step height along the boundary of the shadow zone. Following 60 min of plasma treatment, the root-mean-square roughness (*R*_*q*_) on the top wall surface, which was obtained by atomic force microscope (AFM), was measured at 5.92 nm, which is similar to that on pristine PET, while the high-aspect-ratio nanostructure was fabricated on the plasma exposure zone with 108.6 nm of *R*_*q*_. As *D* increases, more oxygen ions can reach the shadow zone under the metal wires. As a result, nanostructures would form not only on the plasma exposure zone but also on the top of the micro-wall as shown in Fig. 2b,c. The step height between the top of the micro-wall and the valley of the exposure zone is reduced from about 3 to 1 μm as *D* increases from zero to 0.8 mm for the 60 min plasma etching duration (Fig. 2d,e), and is then further lowered less than 0.1 μm for *D* = 2.0 mm (Fig. 2f), indicating the height of micro-wall could be controlled as a function of the gap distance.

The morphology of the top surface of the micro-wall also varied with respect to the gap distance. For *D* = 0.8 mm, the widths of the nanostructures were similar on both the exposure and shadow zones (≈60 nm), but the roughness varied from 63.3 nm on the top surface of the micro-wall to 93.8 nm on the exposure zone. This is because there might be fewer total plasma radicals exposed to the shadow zone than in the exposure zone. With *D* = 2.0 mm (Fig. 2f), the nanostructures were developed uniformly, while the micro-wall pattern is hardly observable in a top view image. The surface profiles were measured in micro and nanoscale, showing a drastic decrease in the step height with increasing *D* (Fig. 2g), while the nanoscale roughness evolves intensively throughout the entire area with the comparable roughness value, as shown in Fig. 2h. The roughness on the top of the micro-wall (shadow zone) was shown to increase from *R*_*q*_ = 5.9 to as high as 87.5 nm as *D* changed from 0 to 2.0 mm, comparable to the value of 89.4 nm measured for the exposure zone.

### Nanoscale etching mask for large area nanostructuring

**Figure 3:**
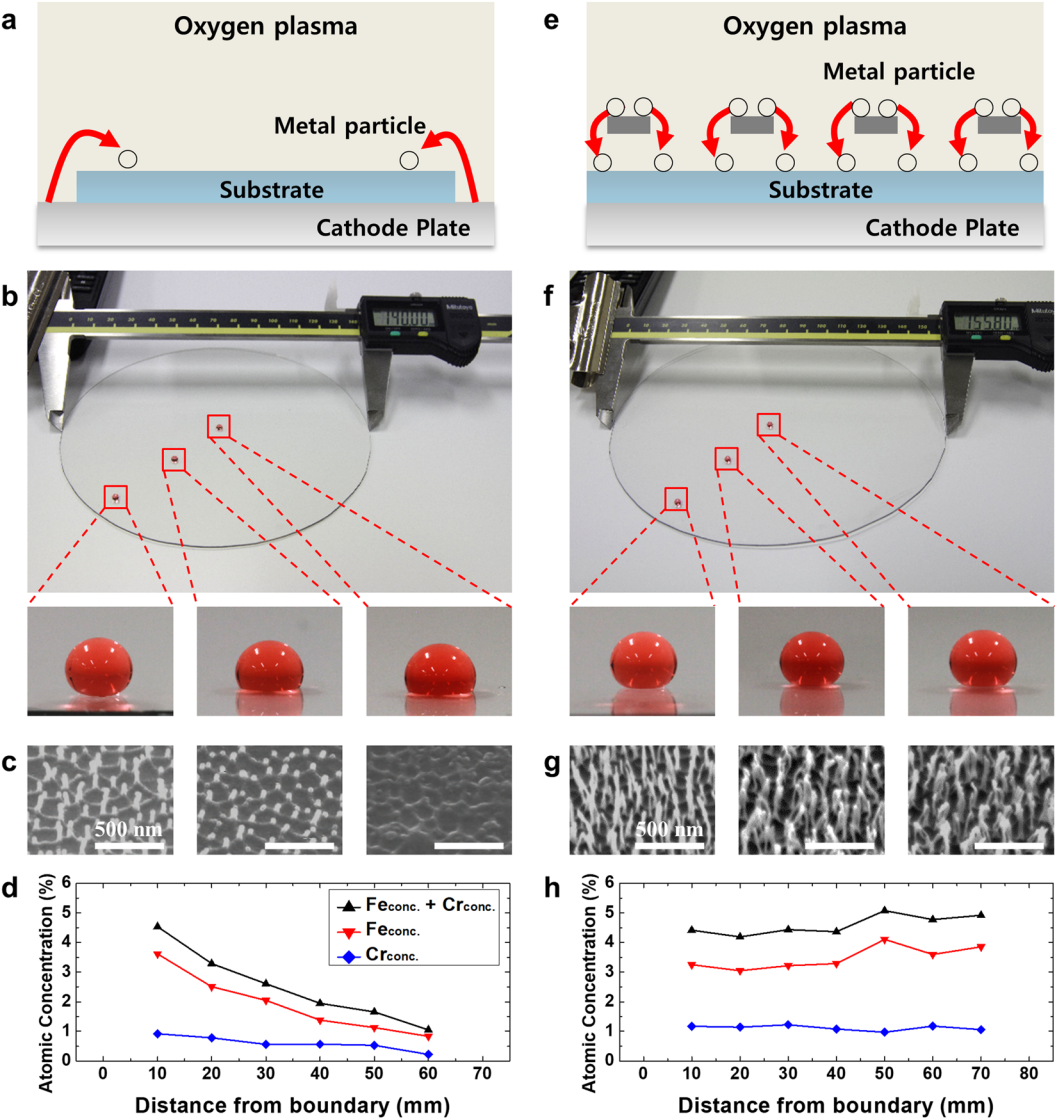
(**a**) Schematic of conventional nanofabrication through O_2_ plasma preferential etching. Metal atoms are sputtered from the stainless-steel cathode plate and co-deposited on the substrate to act as a nanoscale etch mask. (**b**) Optical microscope images of water droplets on the three different spots on the plasma-treated PET substrate with a diameter of 140 mm. (**c**) SEM images of the three different spots on the substrate. (**d**) The atomic concentration of metal compounds on the plasma-treated substrate according to distance from the boundary. (**e**) Schematic of novel fabrication method assisted by metallic mesh overhung with *D* = 2 mm. Metal atoms are sputtered from the overhung mesh. (**f**) Water droplets with high CA on the three different spots on the plasma-treated PET substrate with a similar diameter of 155.8 mm. (**g**) SEM images of the three different spots on the substrate. (**h**) The atomic concentration of the metal compound on the plasma-treated substrate.

As mentioned previously, plasma-based nanostructuring of the polymeric substrate has been used to form nanostructures with a high aspect ratio through the selective ion etching mechanism. Figure 3a–d show the nanostructure formation through conventional plasma-based selective ion etching on a PET sheet placed on a stainless-steel cathode plate. The metal atoms are sputtered from a cathode plate with a diameter of 160 mm by oxygen ions, and are then redeposited on PET to serve the essential role of nanoscale etching masks for nanostructuring (Figs. 1b and 3a)^29^. However, since sputtering the metallic components by oxygen plasma only takes place on the outer region of the cathode plate where PET is not covered, the co-deposition rate of sputtered metallic atoms would be reduced with the distance from the boundary. Figure 3b shows the results of 30 min of O_2_ plasma-treated PET substrate with a diameter of 140 mm, on which the water CA was measured to decrease from 155 to 100° with the distance from the boundary. The non-uniform nature of the CA can be explained by the scanning electron microscope (SEM) images shown in Fig. 3c, which reveals that nanoscale surface features were more prominent on the part near the boundary of the substrate, which is close to the uncovered region on the cathode plate, while the center of the substrate revealed smoother roughness with no clear nanoscale features. The height of the nanostructures was reduced from 204 to 21 nm from the boundary to the center, respectively. X-ray photoelectron spectroscopy (XPS) analysis on several different positions from the metal source was conducted for the quantitative measurement of metal compounds. The atomic concentrations of Fe and Cr, which are the main components of the stainless-steel cathode plate, were evaluated in terms of the distance from the boundary of the PET substrate, revealing that the concentrations of both Fe and Cr were gradually decreased as the distance from the boundary increased (Fig. 3d). Therefore, as the center of the PET disc suffered from a lack of metal co-deposition, the height and distribution of the high-aspect-ratio nanostructures hardly managed to form uniformly over the large area. It has been reported that the concentration of metallic compounds on the polymeric surface corresponds to the roughness of the nanostructured surface^32^. As the size of the substrate is relatively larger than the cathode plate, the sputtered atoms, serving as the nanoscale etching mask, cannot reach the entire region on the target substrate. As a result, it can be considered that even though the conventional selective ion etching method is shown to be very powerful in forming nanostructures on polymers or Si, it reveals obvious limitations in forming the well-distributed nanostructures over the larger surface area, as the size is comparable to that of the cathode plate. In comparison, the proposed method with a dual scale etching mask shows well-distributed nanostructures over the entire substrates, as shown in Fig. 3e–h. During the O_2_ plasma treatment, the metal sputtering occurs on the overhung metal mesh, and sputtered metal atoms are uniformly co-deposited on the PET surface placed below the mesh with *D* = 2 mm (Fig. 3e). Fig. 3f shows the results of 30 min of O_2_ plasma-treated PET substrate with a diameter of 155.8 mm, which is similar to the diameter of the cathode plate in order to cover the entire cathode plate. As shown in the SEM images presented in Fig. 3g, high-aspect-ratio nanostructures were well fabricated throughout the entire area of the PET substrate. Note that the aspect ratio (the height over the width) of the nanostructures is uniform at 12.4 and that the distribution (or number density) was similarly uniform regardless of the location. The microscale step was not clearly observed since the gap distance is sufficiently large for the plasma radicals to reach the entire surface, indicative of the reduced microscale shadowing effect for *D* = 2.0 mm. After hydrophobic coating, the surfaces show high water CA of 155° as well as roll-off behavior of water droplets (see Supplementary Movie S1), regardless of the distance from the boundary. XPS analysis confirmed that the total concentrations of Fe and Cr co-deposited on the substrate were uniformly distributed between 4.2 to 5.1 wt.% across the whole surface.

### Tunable wettability on a nanostructured surface

**Figure 4:**
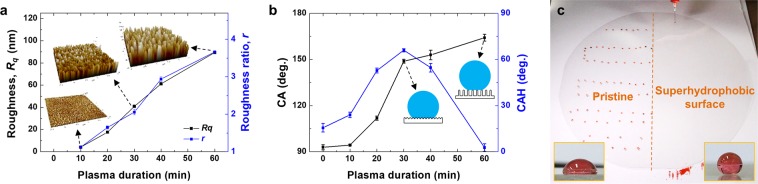
(**a**) Roughness values (*R*_*q*_ and *r*) of plasma-treated PET surface according to plasma duration when *D* = 2.0 mm. (**b**) Water CA of PET surface after plasma etching and hydrophobic thin-film coating. (**c**) Comparison of wettability between the pristine and superhydrophobic PET substrate with a diameter of 500 mm.

Fig. 4a shows that the roughness (*R*_*q*_) of the PET surface after O_2_ plasma etching with *D* = 2.0 mm. The *R*_*q*_ tends to increase almost linearly with the duration of plasma treatment. Until 10 min of plasma treatment, the concentration of metal compounds from the co-deposition of sputtered atoms is not sufficient to form metallic clusters that act as a nanoscale etch mask, so the surface roughness is maintained at a low level of few nm. With increasing plasma treatment duration over 10 min, nanoscale bumps with a low aspect ratio evolve into nanopillar or nanohairy structures through further anisotropic etching, which is induced by the nanoscale etch masks^29,32^. The change in morphology of the nanostructure leads to roughness (*R*_*q*_) increment to 40.9, 61.2, and 89.4 nm for the plasma treatment of 30, 40, and 60 min, respectively. With subsequent hydrophobic thin-film deposition by plasma polymerization of hexamethyldisiloxane (HMDSO) on the nanostructured surface, the CA is increased to 148.8 ± 1.2°, 152.9 ± 3.1°, and 164.1 ± 2.2° for the plasma duration of 30, 40, and 60 min, respectively. In addition, the morphology of the nanostructure significantly affected the adhesion of the water droplet as estimated by measuring the contact angle hysteresis (CAH) which presents the difference between the advancing and receding CAs. As shown in Fig. 4b, the CAH value gradually increased from 15.6° (the intrinsic value of HMDSO coating on a flat smooth surface) to 66° with increasing plasma treatment duration up to 30 min. It then rapidly decreased to 2.7° after longer duration of plasma treatment (60 min). This transition can be explained by the conventional theories presented by the Wenzel (WZ) model and the Cassie-Baxter (CB) model^35^. When a solid surface has low roughness either in micro- or nanoscale, a water droplet has a conformal contact with the solid surface covering grooves between protrusions, which is generally referred to as the WZ state^35^. The apparent CA can be described with the following equation:

1 $$\cos \,{\theta }_{app}=r\,\cos \,\theta $$

where *θ*_*app*_ indicates the apparent contact angle predicted by the WZ model, $r$ is the non-dimensional roughness ratio defined as the ratio of the actual surface area to the projected surface area, and *θ* is the intrinsic CA measured on a flat smooth surface. However, as the aspect ratio of nanostructure increases with further plasma treatment up to 60 min, the water only partially wets the top parts of roughness due to the air trapped between nanostructures beneath the water droplet, forming a CB state. The apparent CA is expressed as follows^35^:

2 $$\cos \,{\theta }_{app}=f\,\cos \,\theta -(1-f),$$

where *θ*_*app*_ is the apparent CA as predicted by the CB model and *f* is the area fraction of the solid surface in contact with the liquid. In contrast to the WZ state (Eq. 1), the CB state is characterized by an extremely low CAH, which causes roll-off of water droplet. In the present experiment, it was found that the CAH decrease to 2.7° while the apparent CA increased to 164.1 ± 2.2° after plasma treatment for 60 min, indicating that the surface exhibited the CB state due to a low area fraction in Eq. (2).

As demonstrated in the previous section (Fig. 3f), the superhydrophobicity can be imposed on the large-scale PET substrate with a diameter of 500 mm. As shown in Fig. 4c, half of the right-hand side on the PET substrate has high-aspect-ratio nanostructures to render superhydrophobicity, while the other half remains untreated flat with a mild hydrophobicity (as shown in the left inset image). The water drops were pinned on the untreated PET surface due to the high CAH value and the low mobility of the water droplet with the original nature of PET. By contrast, on the right side of the sample which has nanostructures, the water droplets easily rolled off due to the high CA (a right inset image), the low CAH value, and the high mobility of the water droplet.

### Superhydrophobicity with the tunable water adhesion on the hierarchically structured surface

**Figure 5:**
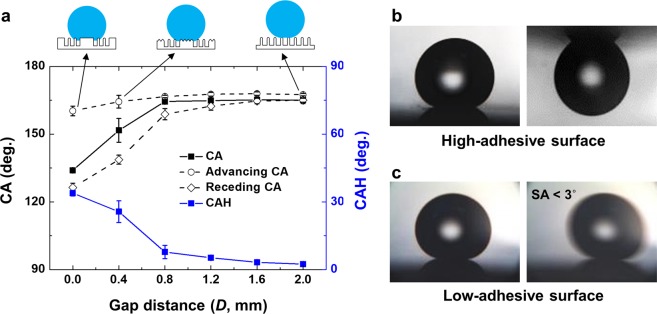
(**a**) Static and dynamic wettability measurements of 60 min of plasma-treated PET substrate with respect to the gap distance. Illustrated insets on the top represent the wetting state for each case. (**b**,**c**) Water droplet adhesion behaviors and morphology illustration of (**b**) high-adhesive surface (*D* = 0.4 mm) and (**c**) low-adhesive surface (*D* = 2.0 mm). The diameter of the droplet in (**b**) and (**c**) is about 2 mm.

The static, advancing, and receding CAs and CAHs were compared as a function of the gap distance (*D*) after 60 min of O_2_ plasma etching. As shown in the plot in Fig. 5a, the water CA is measured to gradually increase as *D* increases, and maintains high (≥164°) when *D* ≥ 0.8 mm and the CAH decreases below 10° (*D* = 0.8 mm) and further down to 2.7° on *D* = 2.0 mm. However, for *D* ≤ 0.4 mm, the CAH was shown to be higher than 20°. This difference in CAH has strong relationships with different surface morphologies implemented by the gap distance control. In the previous section, we found that the height of the micro-wall and the morphology of the nanostructures formed on top of the micro-wall varied as a function of the gap distance, while the shape of the nanostructure formed in the area directly exposed to the plasma was not significantly changed. In this case, the wetting model can be represented as a hybrid form on the micro-wall (shadow zone) and on the nanostructure (exposure zone). With *D* ≤ 0.4 mm, the nanoscale roughness (*R*_*q*_) on the micro-wall is low due to limited ion reaching, while that on the exposure zone is high, rendering WZ and CB states on the shadow zone and exposure zone, respectively. With this hybridized wetting regime, the TCL of water drop can be semi-continuous along with the top of the micro-wall, rendering the WZ regime due to low-aspect-ratio nanostructures. However, the exposure zone has high-aspect-ratio nanostructures, rendering the CB regime and discontinuous TCL. This hybridized regime of WZ and CB states results in the superhydrophobicity with high adhesion. With *D* = 2 mm, in contrast, the top surfaces of the micro-wall and the exposure zone showed similar roughness values of 87.5 and 89.4 nm, respectively, indicating that all surfaces had superhydrophobicity with CB state. Thus, this nanostructured surface with a high-aspect-ratio on the entire area renders the superhydrophobicity with low adhesion.

As noted previously, the hybrid surface for *D* = 0.4 mm presents high water adhesion due to the conformal contact of the water drop pinned to the micro-wall network, so that it can even be turned upside down, as shown in Fig. 5b. By contrast, the water drop on the hierarchical surface of the CB condition for *D* = 2.0 mm is found to roll off easily, even at a very low tilting angle less than 3°, as shown in Fig. 5c (denoted by the low-adhesive surface). Overall, it can be concluded that the water adhesion on the superhydrophobic surfaces is controlled by the gap distance. The high-adhesive hybrid surface is prepared at *D* = 0.4 mm with a CAH of 23.2°, while the low-adhesive hierarchical surface is prepared at *D* = 2.0 mm with an extremely low CAH ≤ 3°.

### Application: Water transport and evaporation control

**Figure 6:**
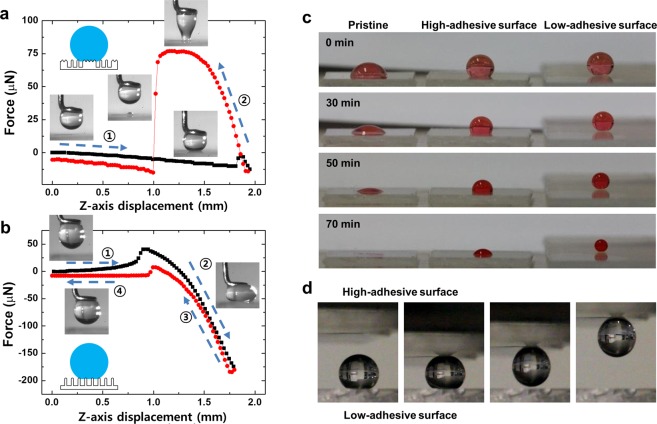
(**a**,**b**) Adhesion force measurement of the water droplet on (**a**) high-adhesive surface and (**b**) low-adhesive surface. Numbers represent the order of measurement procedure; the balance stage moved upward until the water droplet contacted the sample, then the stage moved downward until the water droplet was no longer in contact with the samples. (**c**) Water droplet evaporation behavior on three different surfaces. (**d**) Water droplet manipulation or transportation using two adhesion tuned surfaces.

The water adhesion values on different surfaces are directly measured with the adhesion force required to detach from each surface using the micro-electromechanical balance system. Figure 6a,b compare the water adhesion between the high-adhesive hybrid surface (*D* = 0.4 mm) and low-adhesive hierarchical surface (*D* = 2.0 mm). The balance stage was controlled to move up until the sample surface contact a water droplet of 2 μL that was attached under the ringshaped tip, then it moved down until the water droplet was completely separated from the surface. For the high-adhesive surface, the water droplet was continuously elongated until it detached from the surface, which implies strong adhesion (denoted as step 2 in Fig. 6a), and the maximum adhesion force was recorded as 76.9 μN. In the other case of the low-adhesive surface, even though the water droplet was pressed with a large force of 175 μN during the stage moving up (denoted as step 2 in Fig. 6b), it was easily detached while quickly recovering its original spherical shape. The adhesion force on the low-adhesive surface was measured to be lower than 7.5 μN, which is about 10 times lower than that on the high-adhesive surface.

These superhydrophobic surfaces with different water adhesion can be adopted for the water droplet evaporation and water droplet manipulation. Recent studies have reported that the evaporation control can be used to control precipitation shape, heat transfer efficiency, and dew condensation rate^18,36,37^. We compared the evaporation behavior of water droplets on three different surfaces of pristine surface, high-adhesive surface, and low-adhesive surface, as shown in Fig. 6c. The volume of water droplets on high-adhesive surface shrinks while maintaining the contact area with the substrate, which represents evaporation behavior similar to that of the droplet on the pristine surface. The CA of the water droplet gradually decreases on the high-adhesive surface until the droplet almost dried after 70 min. By contrast, in the case of a low-adhesive surface, the volume of the water droplet shrinks while maintaining a spherical shape, as well as the CA of higher than 150° until 70 min after deposition, showing robustness in extreme wettability. The main reason for the difference between these two evaporation behaviors is the pinning of the water droplets onto the contact region of the water droplet. The hybrid high-adhesive surfaces form the continuous TCL, even at high CAs, from covering the surface cavities conformally by water^18,21–23^, resulting in the contact line hardly receding by the pinning of the contact line along the surface of the WZ wetting micro-wall. On the other hand, as the TCL becomes discontinuous on the low-adhesive surfaces, resulting in reduced water contact area in micro or nanoscale^19,20^, the water droplets can easily evaporate while retaining their spherical shape due to the receding of the contact line without pinning during evaporation in the steady CB wetting state. The adhesion tunable superhydrophobic surfaces can also be applied for the transportation of water droplets. As shown in Fig. 6d, when the high-adhesive surface makes soft contact with the water droplet placed on the low-adhesive surface, the water droplet can be easily transported to the high-adhesive surface due to the large contrast in the water adhesion, as measured in Fig. 6a,b. Due to the high adhesion of the upper surface, only a small contact area is sufficient to lift the water droplet out of the lower surface. Based on the results on the water evaporation and transportation, these adhesion tunable superhydrophobic surfaces can be selectively applied in water harvesting^38^, droplet-based biosensor^39^, and crystal growth control^36^.

### Large area low reflective surface

**Figure 7:**
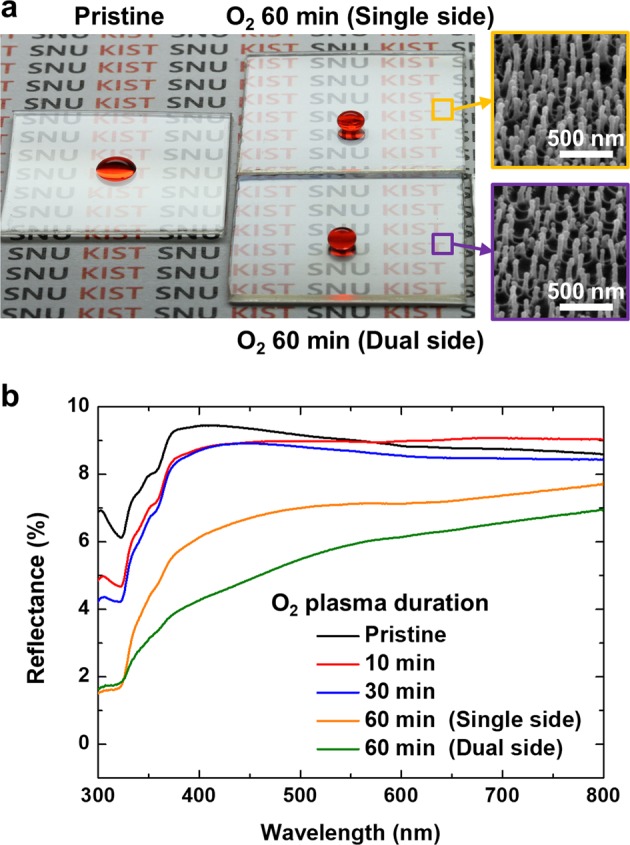
(**a**) Optical image of three PET substrates; pristine with mild hydrophilicity and high reflectivity, and the nanostructured PETs for single side and dual sides showing superhydrophobicity and low reflectivity. Right SEM images indicate that high-aspect-ratio nanostructures are uniformly fabricated on plasma-treated PET substrates. (**b**) Optical reflectance of PET samples measured by UV-vis with respect to plasma treatment condition.

The proposed method enables the uniform distribution of micro/nanostructures over a large area if the metallic mesh is sufficiently large to cover the substrate. It can be used to fabricate low reflectance surfaces for window or optical devices, as well as improve solar cell efficiency^40–42^. When the surface feature has a similar or lower scale as the wavelength of visible light, the refractive index of the solid becomes lower according to solid fraction reduction, so light reflection can be decreased^6,8,43,44^. This phenomenon is known as the moth-eye effect, and many studies have attempted to mimic the nanostructures existing on the moth-eye surface. As shown in Fig. 7a, the hydrophobic film-coated surface with uniformly formed nanostructures with a width of 39.6 nm and an average height of 415 nm at *D* = 2.0 mm presents superhydrophobicity and low optical reflectance. In order to further investigate the effect on the anti-reflectivity, we measured the optical reflectance using UV-Vis with the range of 300–800 nm of wavelength. In the case of the pristine PET, the surface has a reflectance of 6.1–9.4% in the visible light range, as shown in Fig. 7b. For a low plasma duration of 10 or 30 min, as the roughness is still low, the reflectance value is slightly reduced as compared to that on the pristine PET. However, the reflectance of the substrate decreased substantially to 1.5–7.8% in the whole wavelength range as the plasma duration increased to 60 min. Furthermore, the reflectance decreased to 1.6–7.0% when the nanostructure was formed on both sides of the sample. As a result, it can be confirmed that the reflectivity could be reduced by forming the high-aspect-ratio nanostructure on the surface over a large area.

## Discussion

A one-step process for the fabrication of hierarchical micro/nanoscale patterns was suggested in the form of plasma-based patterning with the dual-scale etching mask. Under O_2_ plasma exposure, the metallic mesh overhung on the substrate with a varying gap distance serves as two characteristic etching masks against plasma exposure on the target surfaces by forming microscale wall structures and nanoscale high-aspect-ratio structures. As the sizes for the plasma shadow zone and exposure zone vary with the gap distance, the combination of nanoscale structures and its surrounding microscale wall network was hybridized to form hybrid or hierarchical structures on PET as large as the size of the cathode plate. After hydrophobic coating, the hybrid structured surface shows superhydrophobicity with high water adhesion, while the hierarchical surfaces showed non-stick along with very low water adhesion due to the air trapped between nanostructures beneath water droplets. Two contrasting conditions on which the adhesion force for the hierarchical surface is 10 times smaller than that for the hybrid surface were demonstrated for the water transport and evaporation tests. The uniform distribution of nanostructures formed in a large area was further demonstrated for low reflectance surfaces, along with robust superhydrophobicity.

As our method can effectively and selectively fabricate hierarchical patterns in multiscale depending on various conditions regarding the overhung metallic mesh, it can be applied in mass production processes, such as the industry scale batch process and the roll-to-roll process, and therefore holds substantial potential for the fabrication of functional clothes for textiles, optical windows for smart devices, or energy harvesting with self-cleaning for solar cells.

## Methods

### Sample preparation

Polyethylene terephthalate (PET, LG Chem., Ltd., Rep. of Korea) sheets with a thickness of 1 mm were used as transparent, polymeric substrates. Prior to plasma exposure, PET sheets were thoroughly cleaned with ethanol and deionized water in order to remove any contamination, then dried using a nitrogen gas blower. O_2_ plasma etching was conducted using a glow discharge of oxygen gas at a radio frequency of 13.56 MHz in two different plasma systems with different cathode’s diameters of 160 and 500 mm. Metal mesh made of stainless steel wire was chosen for the dual-scale etching mask. The materials or geometries, such as the diameter of metallic wire and space between two adjacent metal wires, are not particularly limited. A mesh with a diameter of the wire (*d*) of 40 μm and a width of opening (*s*, the space between the metal wires) of 56 μm was chosen for this work, which corresponded with 250 mesh in the ASTM sieve chart. (ASTM Standard E11, 2017).

The metallic mesh was settled directly above the PET substrate by inserting a spacer between the PET and metallic mesh, which was used to tune the gap distance (*D*) between mesh and PET sheet from 0 to 2.0 mm. The vacuum chamber was evacuated to a base pressure of less than 1 mTorr, and the operating pressure was set to 20 mTorr with an oxygen gas flow rate of 20 sccm. The plasma duration was varied from 10 to 60 min at a negative bias voltage of −400 V_b_. After O_2_ plasma etching, the metal mesh was removed, and the hydrophobic thin-film was subsequently deposited on the PET substrate by the plasma polymerization of hexamethyldisiloxane (HMDSO) for 10 sec at −400 V, measured at a thickness of 5 nm.

### Surface morphology observation

The morphology of the O_2_ plasma-etched PET surfaces was observed with a scanning electron microscope (SEM, FEI, Inspect F, Oregon, US) at a 10 kV electron acceleration voltage. In addition, the surface topography was measured with an atomic force microscope (AFM, Park systems Co., XE-70, Rep. of Korea) in a non-contact mode. The root-mean-squared roughness (*R*_*q*_) and roughness ratio (*r*, the ratio between interfacial and projected areas) were obtained and averaged from the measurement results collected from six different spots on each sample.

### Surface chemistry analysis

Surface chemical analysis for the pristine and plasma-treated samples was performed using X-ray photoelectron spectroscopy (XPS, PHI 5800, ESCA System). An Al K_α_ (1486.6 eV) X-ray source was used as the excitation source, and the anode was maintained at 250 W, 10 kV, and 27 mA at a chamber pressure of 2.67 × 10^−8^ Pa with a beam spot size of 400 × 400 μm^2^. The peak position was calibrated using the C1s peak at 284.6 eV.

### Wettability measurement

The wettability of the plasma-treated PET surfaces was measured for the CA and contact angle hysteresis (CAH) with the DI water droplets. For the CA measurements, droplets with a volume of 5 μL were gently deposited on the substrates using a micro-syringe. The CAH was calculated as the difference between the measured advancing and receding CAs. All measurements were taken using a goniometer (Rame-Hart, New Jersey, US) in ambient air at 20 °C with a relative humidity of 25–40%.

### Adhesion force measurement

The adhesive force between the water droplets and the patterned PET surface was measured using a high-sensitive micro-electromechanical balance system (DCAT 11, Data-Physics, German). The patterned PET surface was placed on the balance stage, then a 4 μL water droplet was appended to a ring-shaped tip above the sample. The balance stage moved upward until the samples on the stage contacted and pressed against the water droplet, then the stage moved downward until the water droplet was no longer in contact with the sample. In both cases, the moving speed of the balance stage was kept constant at 0.05 mm/s. During the measurement, the adhesion behavior of a water droplet was recorded with a high-speed camera (APX-RS, Photron, Marlow, UK) at a rate of 500 frames per second.

### Optical reflectance measurement

The optical reflectance of the patterned PET surface was measured using a UV-Vis spectrophotometer (Lambda 20, Perkin-Elmer, MA, USA) in a wavelength range of 300–800 nm at room temperature.

## Supplementary information

Supplementary information.
